# Emerging Biomarkers and Metabolomics for Assessing Toxic Nephropathy and Acute Kidney Injury (AKI) in Neonatology

**DOI:** 10.1155/2014/602526

**Published:** 2014-06-11

**Authors:** M. Mussap, A. Noto, V. Fanos, J. N. Van Den Anker

**Affiliations:** ^1^Department of Laboratory Medicine, IRCCS San Martino-IST, University Hospital, National Institute for Cancer Research, Largo Rosanna Benzi 10, 16132 Genoa, Italy; ^2^Department of Pediatrics and Clinical Medicine, Section of Neonatal Intensive Care Unit, Puericulture Institute and Neonatal Section, Azienda Mista and University of Cagliari, 09042 Cagliari, Italy; ^3^Division of Pediatric Clinical Pharmacology, Children's National Medical Center, Washington, DC 20010, USA

## Abstract

Identification of novel drug-induced toxic nephropathy and acute kidney injury (AKI) biomarkers has been designated as a top priority by the American Society of Nephrology. Increasing knowledge in the science of biology and medicine is leading to the discovery of still more new biomarkers and of their roles in molecular pathways triggered by physiological and pathological conditions. Concomitantly, the development of the so-called “omics” allows the progressive clinical utilization of a multitude of information, from those related to the human genome (genomics) and proteome (proteomics), including the emerging epigenomics, to those related to metabolites (metabolomics). In preterm newborns, one of the most important factors causing the pathogenesis and the progression of AKI is the interaction between the individual genetic code, the environment, the gestational age, and the disease. By analyzing a small urine sample, metabolomics allows to identify instantly any change in phenotype, including changes due to genetic modifications. The role of liquid chromatography-mass spectrometry (LC-MS), proton nuclear magnetic resonance (^1^H NMR), and other emerging technologies is strategic, contributing basically to the sudden development of new biochemical and molecular tests. Urine neutrophil gelatinase-associated lipocalin (uNGAL) and kidney injury molecule-1 (KIM-1) are closely correlated with the severity of kidney injury, representing noninvasive sensitive surrogate biomarkers for diagnosing, monitoring, and quantifying kidney damage. To become routine tests, uNGAL and KIM-1 should be carefully tested in multicenter clinical trials and should be measured in biological fluids by robust, standardized analytical methods.

## 1. Introduction

In neonatology, the evaluation and the monitoring of kidney function continues to be a complex, intriguing, and interesting medical investigation involving the close cooperation of several specialists belonging to pediatric critical care, neonatology, neonatal nephrology, obstetrics, radiology, and laboratory medicine. Drug-induced nephrotoxicity plays an important role in the high prevalence and incidence of neonatal acute kidney injury (AKI), which in turn is the most important cause of morbidity and mortality in preterm babies admitted to neonatal intensive care units (NICUs) [1], especially in those conditions characterized by the absence of oliguria [2]. In fact, the immature preterm kidney with ongoing nephrogenesis is likely to be vulnerable to the hemodynamic changes associated with preterm birth. The early stages of toxic nephropathy and AKI are commonly characterized by very few, nonspecific clinical signs and by nonsignificant variations of conventional serum and urine biomarkers. During toxic nephropathy, the renal functional reserve may mask parenchymal lesions, as estimated by urinalysis, glomerular filtration rate (GFR), blood urea nitrogen (BUN), and serum creatinine (SCr), up to the point where over 75% of the functioning nephrons have been lost [3, 4]. Accordingly, these factors measure incipient kidney failure and in most cases, the finding of normal results does not mean the absence of kidney dysfunction. These drawbacks call for new methods, namely, biomarkers that can identify early and accurately kidney damage and impairment, avoiding the risk of neonatal death and of complications in childhood and adulthood.

## 2. Next Generation Biomarkers for Toxic Nephropathy and AKI

**Figure 1:**
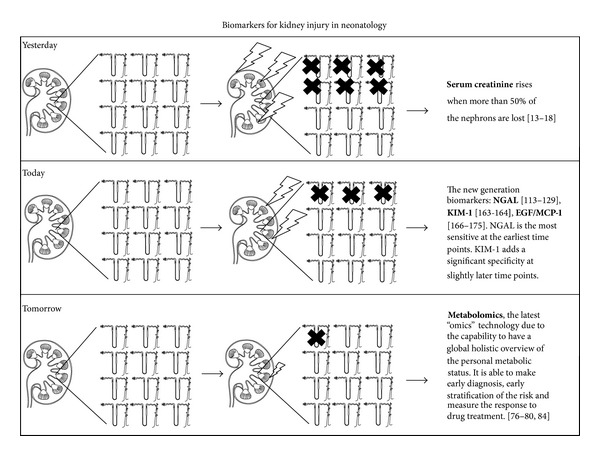
Schematic progresses in knowledge on biomarkers for kidney disease and damage. Abbreviations: NGAL = neutrophil gelatinase-associated lipocalin; KIM-1 = kidney injury molecule-1; EGF/MCP-1 = epidermal growth factor/monocyte chemotactic peptide-1 ratio.

Identification of novel drug-induced toxic nephropathy and AKI biomarkers has been designated as a top priority by the American Society of Nephrology. The concept of developing a new toolbox for earlier diagnosis of disease states is also prominently featured in the National Institute of Health (NIH) Road Map for biomedical research. In 2007, the Acute Kidney Injury Network (AKIN), a collaborative group of investigators from all major critical care and nephrology societies, proposed a staging system based on 3 categories (mild, moderate, and severe) in a way similar to those (risk, injury, and failure) used by the RIFLE staging system. In children, A modified pediatric RIFLE (pRIFLE) classification was proposed in which similar criteria were used for pediatrics [5]. Despite these working classification systems, the diagnosis of AKI is problematic, as current diagnoses rely on two functional abnormalities: functional changes in serum creatinine and oliguria. Both of these are late consequences of injury and not markers of the injury itself. The increasing application in clinical practice of the so-called “omics,” especially metabolomics, seems to offer new attractive perspectives for improving neonatal outcome and management in kidney disease and, more extensively, in critically ill newborns (Figure 1).

## 3. Kidney Development in the Perinatal Period

Human kidney development involves two basic processes: morphologic formation and, ultimately, the acquisition of function. The first one occurs exclusively in utero from the 6th to the 36th week of gestation, whereas the second one starts during the fetal life and accelerates after birth to reach adult levels. In preterm newborns, postnatal renal development exhibits accelerated maturation with a reduced width of the nephrogenic zone, reduced percentage of immature V-stage glomeruli, and increased number of glomerular generations [6]. Immaturity worsens the natural neonatal kidney vulnerability to ischemic and hypoxic insults, mainly caused by higher perfusion rate, and vulnerability to potentially endogenous- or exogenous-toxic substances that may be present in the circulation (drugs, bilirubin, etc.) [7]. Immaturity of renal tubular cells might involve the expression of transporting molecules, the regulation of transporting systems, and the way by which different tubule segments interact. The physiological renal immaturity cannot be considered a risk factor for healthy full-term infants fed an appropriate diet; however, it becomes a major risk in extremely low and very low birth weight (ELBW and VLBW, resp.) preterm infants, often affected by various systemic diseases (dehydration, congestive heart failure, systemic inflammation and sepsis, abrupt changes in intrarenal hemodynamics, etc.) and by inappropriate losses, mechanical ventilation, and exogenous pharmacologic stress. Multiple factors may play a role in the epigenetic modulation of kidney development, including maternal diet, stress and hypertension, drugs administered to the mother or to the newborn, prematurity, low birth weight (LBW), and intrauterine growth retardation (IUGR) [8, 9]. All these factors may lead to a disturbance of nephrogenesis, resulting in low nephron numbers at birth, which may represent the main factor favoring the development of hypertension and, eventually, of end stage renal disease (ESRD) in childhood or adulthood [10]. Finally, the considerable interindividual variability in kidney maturation, recently confirmed by autopsy studies in preterm infants, represent a major risk factor of progressive renal disease in adulthood [11, 12].

## 4. Conventional Biomarkers of Drug-Induced Toxic Nephropathy and Acute Kidney Injury

The current diagnosis of drug-induced nephrotoxicity and AKI relies on a marker of steady-state kidney function, muscle-derived SCr. Unfortunately, neonatal age is typically marked by a non-steady-state condition and even AKI itself represents a very unstable pathological condition. Therefore, SCr becomes a retrospective, insensitive, and even deceptive measure of kidney injury [13–15]: retrospective because SCr concentration may result in a very delayed signal even after considerable kidney injury, it must accumulate over many days, a length of time that is regulated by extrarenal modifiers such as muscle mass and diet [16]; insensitive because as much as a 50% loss of renal function may be required to elevate SCr enough that it comes to medical attention, whereas levels that fall short of this threshold are usually dismissed, despite their known association with excess mortality and prolonged hospitalization, and as SCr is affected by tubular secretion and systemic production, changes in SCr concentration are not specific to tubular injury; deceptive because SCr level often reflects transient physiologic adaptations to volume changes or the presence of chronic kidney disease (CKD), rather than AKI. Most importantly, the measurement of SCr does not identify the cell type that is acutely injured, even though this localization determines the natural history of the disease and its response to therapy. Because small changes in SCr are associated with short- and long-term adverse events, as demonstrated previously [17], determining whether the increase in SCr represents structural damage or a reversible functional change takes on some urgency, as therapeutic strategies are somewhat different [18]. BUN is also widely used for evaluating kidney function; however, likewise SCr, BUN is not a reliable surrogate biomarker of kidney injury because various factors may affect its concentration. For example, an increase in BUN concentration can be found with volume depletion in the absence of any tubular injury. Furthermore, BUN increases concomitantly with the increase of urea synthesis, as occurs with endogenous (catabolic states or blood in gastrointestinal tract) or exogenous (protein supplementation) protein loads [19].

## 5. The Omics Era and Its Impact on the Study of Neonatal Kidney Diseases

With the latest advances in high-throughput technologies, the pace of advances in the “omics” fields that are relevant to clinical medicine has markedly accelerated [20]. The widespread availability of enabling technologies such as functional genomics and proteomics has accelerated the rate of novel biomarker discovery and therapeutic targets for kidney diseases [21]. For example, great attention has been focused on the study of genetic changes contributing to specific renal pathology that could lead to CKD, such as IgA nephropathy and idiopathic membranous nephropathy [22]. Genomics, proteomics, and metabolomics, when taken together as a whole, provide a comprehensive framework, also referred to as systems biology that describes the biochemical function of an organism and its response to challenges. The advent of the microarray, or cDNA chip, allows investigators to search through thousands of genes simultaneously, making the process very efficient. Such gene expression profiling studies have identified several genes whose protein products have emerged as CKD and AKI biomarkers [23, 24]. However, known gene polymorphisms explain only a fraction of associated risk, suggesting that sequence variations in the human genome are only part of the puzzle leading to the evolution of the nascent field of epigenetics [25]. A large number of epidemiology studies suggest that the environment is a major factor in disease etiology [26, 27]. Epigenetics refers to heritable modifications in gene function without alteration of DNA sequences [28]; concisely, epigenetics changes regulate gene expression [29]. The best-known examples of epigenetics modification are DNA methylation and chromatin remodeling by modification of histone proteins [30]; these modifications are potentially reversible and are not associated with changes in DNA sequence [31]; furthermore, they specify functional outputs from the DNA template and are often heritable through cell division [32]. The unifying theme of epigenetic disease is a disruption of normal phenotypic plasticity [33]. Epigenetics alterations are involved in the pathogenesis and progression of kidney disease, especially because these alterations are easily promoted by the plethora of coexisting metabolic alterations and inflammation associated with CKD [34]. Recent reports of epigenetics mechanisms in renal injury, fibrosis, inflammation, and metabolic memory have set the stage for future research in this area [35]. Advancing technologies have radically improved the speed and precision of identifying and measuring proteins in biological fluids, and proteomic approaches are also beginning to yield novel biomarkers for assessing kidney damage [36]. Proteomics can be operationally defined as a field of study that is focused on the identification of proteins, peptides, or their interactions and posttranslational modifications [37]. Clinical proteomics is currently conducted to detect or select biomarkers of disease; mass spectrometry (MS) is the central analytic technique used for most investigational proteomics [38]. In the early 2000's it was introduced as the concept of protein profiling: the fusion of MS technique with pattern recognition, where specific peak profiles, without knowledge of individual peak identity, were treated as biomarkers [39]. In particular, matrix-assisted laser desorption and ionization time-of-flight (MALDI-TOF) and surface-enhanced laser desorption and ionization time-of-flight (SELDI-TOF) MS can profile proteins of low molecular weight (LMW) as well as the metabolic products of serum proteins, originating the so-called peptidome [40]. Briefly, proteinases generate biomarker fragments and circulating protein fragments generated in the diseased tissue microenvironment may serve as diagnostic protein markers. Research studies on the peptidome revealed an apparent abundance of LMW proteins and peptides that potentially contain disease-specific information and showed that changes in the expression patterns of these molecules may be disease specific. Peptidome is a promising high-throughput approach for identifying new potential biomarkers in various body fluids; in particular, urinary peptidome profiling with high-throughput methods such as MB-MALDI-TOF MS or SELDI-TOF MS appears to be a promising tool in nephrology research [41]. Several research papers have demonstrated that “urinary peptidome” may be a resource at least as dynamic and informative as the “urinary proteome” [42, 43].

## 6. Metabolomics for Managing Neonatal Kidney Disease

Genomics, transcriptomics, and proteomics identify genotype and phenotype. On one hand, the genotype of a patient defines the risk or probability of reacting to a disease, drug, or environmental challenge in a certain way; genotype can be considered “static.” On the other hand, the phenotype more closely reflects clinical reality at any given moment, and it may be considered “dynamic.” The advent of metabolomics, in which all of the metabolites in a given tissue or biological fluid are examined (with the caveat that some metabolites will not be detected in any given experiment), is one of the latest advances in the field of omics. The mRNA over- or underexpression (identified as transcriptome) translates directly into corresponding up- or downregulated expression of proteins (surrogate biomarkers), respectively. However, changes in the transcriptome are not necessarily associated with changes in signal transduction and cell biochemistry; therefore, downstream confirmation by analyzing protein concentrations and/or metabolites is commonly performed [44]. In turn, variations of protein levels in biological fluids, cells, and tissues may also not necessarily translate into changes in cell biochemistry and function, since protein expression is not always correlated with activity. Main causes include reaction with oxygen radicals, changes in translational modifications, and allosteric regulation by substrates, products, and other inhibitors and activators. Metabolomics offers several advantages over genomics, transcriptomics, and proteomics, making it extremely attractive for research and clinical purposes. Firstly, metabolites vary both quantitatively and qualitatively at any given time, and this is of great interest, because in most cases pathophysiological pathways and histological damages are directly caused by cell metabolism. Secondly, while transcriptomics and proteomics may be considered “late signals” since their response to a challenge may take hours, days, and sometimes weeks, metabolic response, on the other hand, can be measured very often within seconds or minutes. Thirdly, transcriptomics and proteomics strictly detect endogenous changes, whereas the metabolome communicates with the environment and is an open system. Last but not least, despite the very high overall number of endogenous metabolites (~100,000), the number of major metabolites relevant for clinical diagnostics and drug development has been estimated at 1,400–3,000 molecules [45], which means less data to manipulate and interpret, being genes (~25,000), transcripts (~85,000), and proteins (>10,000,000) greatly outnumbered. It is reasonable to argue that metabolomics is typically more closely associated with a disease process or drug effect than proteins, mRNA, or genes [46].

At first, metabolomics was defined as “the quantitative measurement of the multiparametric metabolic response of living systems to pathophysiological stimuli or genetic modification” [47]. More recently, the same authors have revised the definition of metabolomics as “a global holistic overview of the personal metabolic status,” or in other word, “a snapshot of the chemical fingerprints that specific cellular processes leave behind” [48]. The metabolome was first defined as “the quantitative complement of all of the LMW molecules present in cells in a particular physiological or developmental state” [49]; more concisely, the metabolome can be considered the phenotype reflecting the epigenetics modifications [50]. Two strategies configure metabolomics studies: the targeted and the nontargeted approach [51]. The latter may be defined as a “nonspecific approach,” investigating all the metabolites (both endogenous and exogenous) detectable in a fluid or tissue; this analysis is focused on capturing as much information as possible, providing a functional fingerprint of the physiological and pathological state of the body. The former is focused on the investigation of several well-defined compounds (e.g., those discovered in a new metabolic pathway); it is only used when the target of a drug or disease process is at least partially understood. Metabolic fingerprinting describes the unbiased analysis of the metabolome by examination of metabolite patterns in different experimental groups with the subsequent classification of these patterns into a fingerprint [52]. Samples can be classified if the metabolite fingerprints differ between groups allowing for sample clustering. Metabolite identification relies on public databases [53]: the human metabolome Data Base (HMDB) is the metabolomic equivalent of GenBank. It is an open access database (http://www.hmdb.ca/) providing reference to nuclear magnetic resonance (NMR) and mass spectra, metabolite disease associations, metabolic pathway data, and reference to metabolite concentrations for hundreds of human metabolites from several biofluids [54, 55].

In most cases, proton nuclear magnetic resonance (^1^H NMR) spectroscopy and MS based assays are used for metabolic fingerprinting [56–59]; these techniques require a well-defined sample preparation [60]. Typically, ^1^H NMR spectroscopy allows for the simultaneous detection of 20–50 metabolites with an analytical sensitivity ranging 1–10 *μ*mol/L [61]; below this cutoff, the detection and quantification of metabolites is still unreliable, although high field NMR spectroscopy and cryoprobes can improve sensitivity [62]. On the other hand, MS is still considered the gold standard in metabolite detection and quantification; depending on the metabolite, the sensitivity of MS is in the picomolar and nanomolar range. However, MS should be coupled to an array of separation techniques including gas chromatography (GC) and liquid chromatography (LC); in addition, MS requires longer analytical time (20–60 min for each sample), extensive sample preparation including derivatization and the limitation to volatile compounds [63]. Other technologies less commonly used for metabolomics are Raman and infrared spectroscopy [64, 65]. Each method has serious drawbacks, such that neither by itself is ideal.

In general, biological fluids are considered highly adequate for metabolomics, because they closely represent quantitative and qualitative variations of phenotypic molecular markers such as metabolites. In neonatal and pediatric nephrology, however, urine is considered the ideal sample, since it is a so-called “proximal matrix,” being closer to (or in direct contact with) the kidney, which is the site of disease or drug effect under investigation [66]. This means that urine metabolome better reflects kidney pathophysiological changes, while metabolome in whole blood, plasma, and serum better reflects systemic changes. Furthermore, urine represents an “open system” by which the body through the elimination of water, ions, metabolic degradation, and harmful or toxic substances regulates important balance, maintaining homeostasis. It is also of importance that the urine metabolome includes the intermediate metabolites, which reflects specific metabolic processes. Finally, urine can be collected easily (a spot sample is adequate) and noninvasively: these aspects are of extreme importance in neonatology, especially for preterm babies LBW. Two conditions are essential to perform metabolomics studies on urine samples: first, urine must be collected in a sterile bag or plastic container, because bacteria metabolism significantly interferes on the urine metabolome. Secondly, urine samples must be frozen at −80°C immediately after collection, until analysis [67].

Metabolomics allows to: (a) identify unknown molecular mechanisms; (b) select molecular markers that can be used for drug discovery, preclinical, and clinical drug development; (c) develop diagnostic tools. Theoretically, metabolomics has a great potential in nephrology for identifying metabolic patterns as markers of kidney function, disease, and injury and for elucidating and monitoring pharmacodynamic and toxicodynamic molecular mechanisms [68]. Interestingly, ^1^H NMR-based metabolomics permits to follow metabolism in different areas of the kidney, which could yield important information about nephrotoxicity [69]. Monitoring renal transplantation and allograft rejection are also promising applications for metabolomics [70, 71]. In the neonate, the continuous, abrupt changes in renal hemodynamics, fluid balance, glomerular and tubular functions, and metabolism due to the developmental transition from fetal to neonatal life make it critical for the analysis of the metabolic profile and the research of new molecules associated with pathological conditions. In particular, the body water content significantly differs between premature babies (85%), infants (75%), and adults (50–60%). In addition, the amount of water in the extracellular compartment is almost double in the newborn compared with that in the adult (40% versus 20%, resp.) [72]. However, metabolomics is opening up new perspectives to improve the management of sick newborns and of VLBW and LBW preterm newborns by providing new metabolic profiles and biomarkers associated with perinatal/neonatal maturational processes and their metabolic background [73–75]. In particular, different urine metabolic profiles were found between 26 full term and 41 preterm babies [76]. Interestingly, the urine metabolome discriminated preterm babies with a GA between 23 and 32 weeks from those with a GA between 33 and 36 weeks. Single metabolites recognizing unambiguously these groups were: hippurate, tryptophan, phenylalanine, malate, tyrosine, hydroxybutyrate, N-acetyl-glutamate, and proline. Furthermore, metabolomics seems to be a valuable tool for investigating the pharmacokinetics and the effectiveness of drugs in neonatology [77, 78]. In a clinical study in 21 children with nephrouropathies compared with 19 healthy controls, it was found that renal and urinary tract malformations are associated with specific urine metabolic profiles never overlapping at least in part urine metabolic profile in healthy controls [79]. Metabolomics may play a key role in perinatology, particularly for searching biomarkers of IUGR. In a group of preterm babies with IUGR diagnosed by ultrasonography during pregnancy, urine metabolic profile revealed an increase in the flux of the urea cycle, amino acid metabolism, glycine, serine, and threonine metabolism [80]; interestingly, it appeared to be associated with a significant increase of myoinositol levels in comparison to the control group (*P* = 0.04). Although the role of myoinositol is still unclear, it may be associated with the development of metabolic syndrome. The metabolic profiles in broncoalveolar lavage fluid (BALF) were recently investigated in 12 preterm babies with respiratory distress syndrome (RDS) during mechanical ventilation and at extubation time point, after surfactant administration [81]. By using the GC-MS technical approach, 25 overexpressed metabolites were identified, including 10 with known molecular structure. Metabolomics has been successfully used for managing pediatric asthma, pneumonia, and bronchiolitis [82]. Metabolomics seems to have the capacity to assess the risk of CKD in adulthood in subjects born with ELBW. By comparing the urine metabolic profile in 19 healthy young adults (mean age 24 y) born with ELBW with that of 13 healthy adults of similar age (controls) born at term appropriate for gestational age (AGA), we found two totally distinct cluster regions of metabolites: the first one associated with controls and the other one with subjects born ELBW [83]. By multivariate analysis, the most important discriminating metabolites between the two groups were N-methylhydantoin, glycine, valine, and glutamine. Metabolites significantly increased in ELBW urine samples were correlated to CKD as well as to the metabolic syndrome. Several studies have attempted to find early diagnostic surrogate biomarkers for a variety of renal diseases as well as to direct personalized therapies; in particular, metabolomics has been applied to the study of uremic syndrome, diabetic nephropathy, AKI, polycystic kidney disease, and kidney cancer [84].

Although individual data sets including genomic, epigenomic, proteomic, and metabolomic information are highly informative, integrating them together offers the exciting potential to answer many long-standing questions. From this point of view, metabolomics should be considered complementary to transcriptomics and proteomics. Therefore, integrative analysis has become an essential part of experimental design in the era of next-generation genomics and is no longer the domain of bioinformatics technicians [85].

## 7. Neutrophil Gelatinase-Associated Lipocalin (NGAL)

On the basis of experimental studies on kidney injury in mouse and other animal models, researchers picked the 10 proteins that were most overexpressed in the kidney for further study. Of those, neutrophil gelatinase-associated lipocalin (NGAL) turned out to be a useful marker. NGAL has emerged as the most promising marker of AKI in a number of clearly defined clinical contexts [86]. Human NGAL also named human neutrophil lipocalin (HNL), lipocalin-2 (*lcn2*) or lipocalin 24p3, siderocalin, *α*_1_-microglobulin-related protein, and uterocalin is a ubiquitous 25-kDa glycoprotein consisting of 178 amino acid residues belonging to the lipocalins family [87]. NGAL binds and transports LMW proteins (ligands) as well as lipophilic substances, including the bacterial siderophore enterochelin from gram-negative bacteria, bacilli bactin from gram positive, and carboxymycobactins from Mycobacteria. When these siderophores are bound to NGAL, iron transfer to bacteria is prevented and growth is blocked [88]. In the course of experimental studies inducing kidney ischemia-reperfusion, it was observed that NGAL has the capacity to attenuate the extent and the severity of renal tissue injury by reducing apoptosis and enhancing proliferation of renal tubules [89]. This effect is due to the iron delivery to proximal tubular cells by NGAL; iron, in turn upregulates heme oxygenase-1, a well-known enzyme that protects tubular cells [90]. NGAL can additionally promote renal tubular formation and might enhance tubule repair after AKI [91].

NGAL was firstly isolated from the supernatant of human activated neutrophils [92]; later, it was evident that infection and inflammation, oxidative stress, cytokines, ischemia, cancer, intoxication, and other conditions leading to cellular necrosis, apoptosis, and death induce the rapid upregulation of NGAL synthesis in epithelial cells of various human tissues, including liver, lung, kidney, and trachea [93, 94]. NGAL is thought to be an acute-phase protein with upregulated expression in different inflammatory conditions as well as in cancer [95]; it has also been suggested that NGAL comprises a critical component of innate immunity to exogenous bacterial infections [96]. In healthy subjects, circulating NGAL is filtered through the glomerulus and is then captured by megalin within the proximal tubule, where it traffics to lysosomes and degrades to a 14-kDa fragment being not recycled [97, 98]. Experimental studies on animal models have definitively demonstrated that the response of the kidney to injury consists of the NGAL mRNA overexpression by distal tubular cells and collecting ducts [99]; similarly, the pivotal role of NGAL in regulating the progression of CKD to AKI was demonstrated [100]. A growing body of evidence indicates that NGAL increases within a few minutes in both serum and urine after an injury of kidney tissue (up to 1,000-fold) and thus it has been widely evaluated in clinical studies for the early diagnosis, monitoring, and risk stratification of AKI and other kidney diseases.

AKI induces a rapid and massive upregulation of NGAL mRNA within the thick ascending limb of Henle's loop and in the collecting ducts, originating the so-called “NGAL renal pool” [101]; the accumulation of NGAL in the distal nephron leads to a significant increase in urine NGAL (uNGAL), which represents the major fraction of kidney tissue-derived NGAL. Simultaneously, AKI induces NGAL mRNA upregulation in the liver, in the lung, and in various distant organs, originating a rapid release of NGAL into the circulation, called “NGAL systemic pool.” Finally, uNGAL may originate both from circulating NGAL and from the distal nephron, and this hypothesis has been recently reported as “two-compartment model of NGAL trafficking during AKI” [102]. In this model, systemic NGAL that is produced in the setting of sepsis or renal disease may serve to limit proximal tubular damage, whereas NGAL synthesized locally in the kidney may exert bacteriostatic effects in the distal urogenital tract. According to this model, changes in uNGAL concentration may better predict AKI than those in plasma, being earlier and more specific.

With the intent to introduce the determination of NGAL in clinical practice, new analytical methods have been developed and optimized in biological fluids; in particular, NGAL can be measured in urine by a reliable and automated method, easily adaptable in an emergency setting [103]. Being NGAL a critical component of innate immunity to bacterial infection, it is also expressed during systemic inflammation and sepsis, and thus it increases significantly in the bloodstream and, in turn, in urine. Moreover, during systemic inflammation and sepsis uNGAL significantly increases because of neutrophils accumulation within the tubular lumen. Consequently, uNGAL can increase (a) as a result of a renal tubular damage; (b) in the course of an acute phase response; (c) as the concomitant presence of sepsis with AKI. How can we distinguish sepsis-induced uNGAL from AKI-induced uNGAL excretion? Three isoforms of human NGAL have been isolated: a 25-kDa monomer, a 45-kDa disulfide-linked homodimer, and a 135-kDa heterodimer consisting of a monomer covalently bound with neutrophil gelatinase, also named matrix metalloproteinase (MMP-9) via an intermolecular disulfide bridge. The NGAL/MMP-9 complex formation seems to protect MMP-9 enzymatic activity from degradation [104]. Neutrophils synthesize the monomer and the homodimer, whereas renal tubular epithelial cells synthesize the monomer and, to some extent, the heterodimer [105, 106]. Therefore, we can speculate that an “ideal” immunoassay capable to distinguish various molecular forms of uNGAL should permit to assess the origin of uNGAL and, ultimately, the pathological process leading to the changes in uNGAL concentration [107]. Unfortunately, this “ideal” immunoassay does not exist; more important, developing AKI in the course of sepsis and developing sepsis in the course of AKI are both dynamic pathological processes in a continuous interaction [108].

NGAL has emerged as a very promising biomarker of kidney injury and damage especially because kidney epithelia express and excrete massive quantities of NGAL within 30 minutes into urine when stressed by ischemia-reperfusion injury, nephrotoxins, sepsis, and chronic progressive changes [109, 110]. These findings have been confirmed in various studies in adults, children, and newborns [111, 112]. A milestone in clinical studies evaluating NGAL as a biomarker for AKI is that of Mishra et al., published in 2005 [113]. In a group of 71 children undergoing cardiothoracic surgery, which represents an excellent model of renal ischemia-reperfusion, the development of AKI in 28% of children was detected by substantial increase in serum and urine NGAL 2 hours after cardiac surgery. Importantly, NGAL detected AKI 34 hours earlier than serum creatinine did. Both urine and plasma NGAL were powerful independent predictors of AKI, with an AUC of 0.998 for the 2-hour urine NGAL and 0.91 for the 2-hour plasma NGAL measurement. A conspicuous number of studies on NGAL for assessing AKI in the course of cardiac surgery have subsequently confirmed the results published by Mishra [114–120]. In a prospective study, uNGAL was measured immediately after kidney transplantation and then for subsequent 3 times every 6 hours [121]. NGAL urine levels significantly differed between patients with delayed graft recovery, patients with slow graft function, and patients with immediate graft function; results clearly showed that uNGAL can be used as an early, noninvasive and accurate predictor of the need for dialysis within the first week of kidney transplantation, confirming a previous similar study performed mainly on children [122]. Urine NGAL has also been shown to predict the severity of AKI and dialysis requirement in a multicenter study of children with diarrhea-associated hemolytic uremic syndrome [123]. The measurement of plasma and urine NGAL seems to be a reliable, predictive biomarker of AKI following contrast administration and in the intensive care setting [124, 125]. In the neonate, uNGAL is detectable at birth, showing a wide range of variability in premature newborns (0.51–2815.7 *μ*g/L), probably because NGAL plays an important developmental role in proliferating nephrons of premature kidneys [126]. Urine NGAL was found to be inversely related to birth weight [126]. The sensitivity of uNGAL in detecting oliguria (used as a surrogate of AKI) was found low (31%) while specificity was 90%, suggesting that babies who do not have clinical indicators such as oliguria would test negative for AKI when using uNGAL as a screening mechanism. Reference ranges for uNGAL were established in 50 VLBW premature babies (2–150 *μ*g/L) by an immunoblot assay employing human NGAL recombinant to create the standard curve [127]. Finally, uNGAL can be considered an early biomarker of sepsis in VLBW newborns, discriminating babies with late onset blood culture positive sepsis from those with single blood culture positive for* S. epidermidis* and from those with negative blood culture treated with antibiotics [128]. Despite the fact that NGAL is emerging as a center-stage player in the AKI field as a novel predictive biomarker, large multicenter studies to further define the predictive role of plasma and urine NGAL as a member of the putative “AKI panel” have been initiated [129].

## 8. Kidney Injury Molecule-1 (KIM-1)

Kidney injury molecule-1 (KIM-1) is a biomarker for renal proximal tubular damage discovered only about 15 years ago [130]. KIM-1 is a type I cell membrane glycoprotein containing, in its extracellular portion, a six-cysteine immunoglobulin-like domain, two* N*-glycosylation sites, and a Thr/Ser-Pro rich domain characteristic of mucin-like* O-*glycosylated proteins [131]. The cytoplasmic domain of KIM-1 is relatively short and possesses a potential phosphorylation site, indicating that KIM-1 may be a signaling molecule; the ectodomain is cleaved by metalloproteinases. KIM-1 is also known as T cell immunoglobulin mucin domains-1 (TIM-1), as it is expressed at low levels by subpopulations of activated T cell [132]; another KIM-1 homolog is an African green monkey protein cloned as hepatitis A virus cellular receptor-1 (HAVCR-1), expressed by hepatocytes [133]. The KIM-1 gene is markedly upregulated in the postischemic rat kidney; a large pharmaceutical company consortium, using an unbiased genomic approach to evaluate genes upregulated with the nephrotoxin cisplatin, determined that KIM-1 was upregulated more than any of the 30 000 genes tested [134].

KIM-1 is a phosphatidylserine receptor on renal epithelial cells that recognizes and phagocytizes apoptotic cells commonly present in the postischemic kidney; this function has the property to transform normal proximal tubule cells into a phagocyte [135]. As a result, KIM-1 is involved in the clearance of the apoptotic debris from the tubular lumen and thus may play an important role in limiting the autoimmune response to injury since phagocytosis of apoptotic bodies is one mechanism for limiting the proinflammatory response [136]. KIM-1 positive atrophic tubules are usually surrounded by fibrosis and inflammation; this association suggests that KIM-1 might be involved in the development of interstitial fibrosis [137]. In normal human and rodent kidney, mRNA and protein are expressed at very low levels [138]; when an injury (e.g., hypoxia and ischemia) affects the kidney, mRNA KIM-1 levels increases more than any other known transcript and the protein is localized at very high levels on the apical membrane of proximal tubule in that region where the tubule is most affected. The cell surface (mature) form of KIM-1 is a 104 kDa peptide. After injury the ectodomain of KIM-1, consisting of a 90 kDa soluble protein (soluble KIM-1), is shed from proximal tubular kidney epithelial cells into urine [139, 140]. Soluble KIM-1 may form a protective layer on the proximal tubular cells, thereby protecting them from protein casts forming within the lumen.* In situ* hybridization and immunohistochemistry revealed that KIM-1 is expressed in dedifferentiated proximal tubular epithelial cells in damaged regions, especially in the S3 segment of the proximal tubule in the outer strip of the outer medulla, a region that is highly susceptible to injury as a result of ischemia or toxins. Because KIM-1 colocalizes with markers of proliferation, it was suggested that KIM-1 plays a role in the regeneration process.

A large number of studies in animal models have provided robust evidences that KIM-1 is expressed in the affected segments of the proximal tubule whenever a toxin or pathophysiological state results in dedifferentiation of the epithelium [141]. Dedifferentiation is a very early manifestation of the epithelial cell response to injury [142]. In particular, KIM-1 induction has been demonstrated after ischemic renal tubular injury and necrosis [143]. KIM-1 is also expressed in other conditions where proximal tubules are dedifferentiated, including toxic nephropathy from cyclosporine [144], cadmium [145], and other toxic compounds [146], and in renal cell carcinoma [147, 148]. In a protein-overload model of tubulointerstitial disease, KIM-1 was found markedly induced within tubular cells and conspicuously excreted into urine [149], suggesting that it is involved in the pathogenesis of proteinuria-induced renal damage/repair and that its urine levels may serve as a marker of proteinuria-induced renal damage. In mice, KIM-1 was found upregulated in polycystic kidney disease especially in regions of the kidney where fibrosis takes place [150]. Extrarenal functions for KIM-1 have been described in the immune system, where the mouse* kim-1* gene is a susceptibility locus for experimental allergic asthma [151], and human KIM-1 (TIM-1) is involved in the regulation of T_H_2 cytokine production. The molecular mechanisms regulating KIM-1 urinary levels have been recently elucidated [152]. KIM-1 shedding can be enhanced dramatically by pervanadate, a potent inhibitor of protein tyrosine phosphatases. The constitutive and pervanadate-induced shedding of KIM-1 is mediated by metalloproteinases and regulated by extracellular signal-regulated kinase (ERK) and p38 mitogen-activated protein kinase (MAPK), respectively. The protein secondary structure in the juxtamembrane region of KIM-1 is important for its cleavage. Ectodomain cleavage of KIM-1 results in generation of a truncated 14-kDa cell membrane-associated and tyrosine-phosphorylated KIM-1 fragment [152].

KIM-1 is a specific histological biomarker for diagnosing early tubular injury in renal biopsies: it was found that positive staining in proximal tubules correlates very well with renal dysfunction, being a very useful biomarker to diagnose kidney epithelial cell injury in renal allografts [153]. When a renal transplant recipient has renal dysfunction without acute cellular rejection detectable in the renal biopsy, negative staining of KIM-1 suggests that renal dysfunction is not associated with tubular injury and may be attributed to prerenal factors. Clinical studies have reported that urinary excretion of KIM-1 is an independent predictor of long-term graft loss and therefore a promising new biomarker in early prediction of graft loss [154, 155]. KIM-1 can be considered an outstanding biomarker for kidney injury for at least three reasons: first, it is not detectable in normal kidney, second it is expressed by the affected segment of the proximal tubule whenever the initial ischemic or toxic insult induces dedifferentiation of the epithelium, and third the ectodomain of KIM-1 is shed from injured cells, being excreted into urine within 12 h and persisting over time before regeneration of the epithelium [156]. Urinary KIM-1 concentration is closely correlated with the severity of kidney injury, representing a noninvasive and sensitive surrogate biomarker for diagnosing, monitoring, and quantifying kidney damage [157]. Due to these superb characteristics, the Predictive Safety Testing Consortium (PSTC), a cooperation group consisting of members from the biotech and pharmaceutical industry together with members from academia, from US Food and Drug Administration (FDA), and from the European Medicines Agency (EMA), has included KIM-1 in the short list of biomarkers under investigation to detect drug-induced nephrotoxicity [158, 159]. KIM-1 ectodomain soluble protein can be measured by a microsphere-based Luminex xMAP technology employing polyclonal antibodies raised against the human KIM-1 ectodomain; this method requires only few microliters of urine sample. The lower limit of detection for this assay is 4 ng/L, and the inter- and intraassay variability, expressed as coefficient of variation (CV, %), is less than 10%. In healthy subjects, urine KIM-1 excretion expressed as mean ± standard deviation (SD) is 58 ± 8.0 ng/day, whereas in untreated patients with nondiabetic proteinuria KIM-1 excretion is 1706 ± 498 ng/day [160]. This microbead technique is an adaptation of the previously described sandwich ELISA assay, which is known to mirror findings by western blot analyses [161, 162].

Very few studies investigated the clinical usefulness of KIM-1 in newborns, infants, and children. In a case-control prospective study performed in 20 children aged 0.16–17 years with severe congenital hydronephrosis (HN) caused by ureteropelvic junction obstruction (UPJO), the urine KIM-1 levels were significantly elevated in subjects developing an obstructed kidney but not yet undergone pyeloplasty [163]. Three months after surgery, the concentration of urine KIM-1 decreased significantly but did not reach the values found in a group of 20 children with dilated but not obstructed kidney (mild nonobstructive HN). Urine levels of KIM-1 were negatively correlated with differential renal function (DRF) assessed by the radionuclide scan. The strong negative correlation between KIM-1 level in the pelvic urine and DRF of the affected kidney confirms that urine KIM-1 ectodomain soluble protein is closely related to tissue KIM-1 and with the severity of renal damage. Recently, urinary KIM-1 was measured in a cohort of 123 premature newborns in a NICU: in 52 babies with GA ≤26 weeks, KIM-1 expressed as geometric mean and 95% confidence intervals, was 226 ng/L (184–277 ng/L). As GA increased, KIM-1 progressively declined, being geometric mean 158, 155, and 143 ng/L in babies with GA ranging between 26–28, 28–30, and 30–36 weeks, respectively [164]. An advantage of KIM-1 over uNGAL is that it appears to be more specific to ischemic or nephrotoxic AKI and is not significantly affected by prerenal azotemia, urinary tract infections, or CKD.

## 9. Opportunity and Challenges for Utilizing Monocyte Chemoattractant Protein-1 and Epidermal Growth Factor as Biomarkers of Kidney Damage and Repair

Progressive CKD involves the impairment of several tracts of the nephron by the activation of pathological processes, specifically glomerulosclerosis, tubulointerstitial fibrosis, and vascular sclerosis. Of these, tubulointerstitial changes are greatly relevant in determining the progression of kidney damage; indeed, the severity of tubular atrophy, interstitial cell infiltration, and fibrosis correlates with the decline of kidney function. Most of cell infiltrates are monocytes and differentiated interstitial macrophages: they play a central role in innate immune protection both early, by a cytotoxic and proinflammatory action, and later, by phagocytizing cellular debris and apoptotic bodies in order to initiate the process of tissue repair [165]. Concomitantly, monocytes and differentiated interstitial macrophages generate radical oxygen species, nitric oxide, complement factors, and proinflammatory cytokines leading to a direct damage to resident cells. Over the past 15 years, several studies both on animal model and on patients with kidney disease have reported encouraging results on the clinical utility of biomarkers overexpressed by renal tubular cells and by monocytes-macrophages infiltrating the peritubular space. In patients with chronic tubulointerstitial injury, the urinary excretion rate of C-C motif chemokine ligand 2 (CCL2), also called monocyte chemoattractant protein-1 (MCP-1) and that of epidermal growth factor (EGF) together with the calculation of the ratio EGF/MCP-1 seem to represent powerful prognostic indexes, opening new perspectives for the early, accurate evaluation of tubulointerstitial injury and repair. In addition, MCP-1 gene activation in patients with kidney injury is reflected by increased urinary excretion of MCP-1 and thus it may be a useful biomarker of AKI, since it mediates acute ischemic and toxic kidney injury, as demonstrated elsewhere [166]. The severity of progressive interstitial fibrosis is strongly correlated with the extent of macrophage infiltration in the peritubular space, which in turn is positively correlated with the expression of chemokines (chemotactic cytokines constituting a large family of peptides classified into four subfamilies). MCP-1 belongs to the CC chemokine subfamily (*β*-chemokine); it is a potent chemotactic factor for monocytes and macrophages. MCP-1 gene is located on chromosome 17 (17q11.2-q21.1) and the mature form of the protein is composed of 76 amino acid residues with a molecular weight of 13 kDa. The major source of MCP-1 is monocytes and macrophages. In biopsy specimens from patients with acute interstitial nephritis, MCP-1 was found clearly upregulated; the gene and the protein expression were primarily localized in tubular and glomerular parietal epithelial cells, as well as in infiltrating monocytes and macrophages [167]. In addition, in patients with immunoglobulin A nephropathy, urinary excretion of MCP-1 was higher than that in healthy subjects and positively correlated with the renal MCP-1 gene expression [167]. On the other hand, human EGF is a 6 kDa peptide consisting of 53 amino acid residues synthesized by the ascending portion of the Henle's loop and the distal convolute tubule. Human EGF is a peptide growth factor inducing epithelial cell growth and metabolism; various experimental and clinical studies have found that EGF acts as a mediator of normal tubulogenesis and tubular regeneration after injury [168, 169]. In a rat model involving neonatal and adult rats with chronic unilateral ureteral obstruction, prolonged administration of EGF attenuated the impairment of renal development in the maturing rat kidney affected by chronic unilateral ureteral increased the proliferation of renal tubular epithelial cells obstruction and suppressed apoptosis [170]. Progressive increase over time in urinary EGF excretion has been demonstrated in asphyxiated babies put on assisted ventilation [171] as well as in the course of therapeutic treatment of children with recurrent urinary tract infection and in those with vesicoureteric reflux [172]. The calculation of the EGF/MCP-1 ratio has been proposed as a better index of the relationship between renal tubular regeneration and interstitial inflammation; as previously demonstrated, an inverse relationship exists between renal gene expression of EGF and MCP-1 [173]. In a cohort of 132 patients with biopsy-proven IgA nephropathy, the urinary EGF/MCP-1 ratio showed a better ability to predict outcomes rather than the two single measures, leading to the conclusion that it may be considered a prognostic index of ESRD: at the cutoff value of 23.2, sensitivity was 88.9% and specificity 86.4% [174]. EGF/MCP-1 ratio was found significantly downregulated in two groups of untreated children with ureteropelvic junction obstruction compared with controls; in addition, surgical treatment of urinary obstruction improved significantly EGF/MCP-1 ratio when compared with the group of obstructive ureteropelvic junction obstruction [175]. On the basis of the current available results from the literature, it is desirable to perform further multicenter trials in order to validate definitively these biomarkers, taking into account the importance to early assess the capacity of repair of the renal tubular cells.

## 10. Conclusions

Recent progress in medical care has contributed to improved survival among all but the most immature infants [176]. In LBW and VLBW the mortality rate continues to be high and AKI plays an important role in reducing survival in these babies [177]. Conventional biomarkers of toxic nephropathy and AKI, such as oliguria, SCr, and BUN, are insensitive and cannot be considered markers of injury. Unfortunately, the management of critically ill newborns is often crucial for the absence of specific symptoms and signs related to kidney impairment and damage. Increasing knowledge in the science of biology and medicine has accelerated the discovery of novel biomarkers and elucidated their roles in molecular pathways triggered by physiological and/or pathological conditions. Emerging tools, like metabolomics, depend on sophisticated technologies (LC-MS, GC-MS, ^1^H NMR, etc.) which play a pivotal role, contributing to the sudden development of new biochemical and molecular tests. There is an urgent need to translate these developing methods (epigenomics, metabolomics, etc.) and next generation biomarkers (NGAL, KIM-1, MCP-1, etc.) from bench to bedside in order to improve clinical outcome and quality of care in acute ill newborns and infants. Metabolomics seems to be a very promising tool minimizing false positive and false negative results. Interestingly, metabolomics may become a powerful tool for reducing health care costs associated with length of hospitalization, appropriateness in drug administration, severe complications, hospital-acquired infections, and so forth. It is likely that NGAL and KIM-1 will emerge as tandem biomarkers of AKI, with NGAL being most sensitive at the earliest time points and KIM-1 adding significant specificity at slightly later time points. This combination may be an excellent opportunity to improve the efficacy of the therapeutic treatment in sick newborns and, in turn to reduce the risk of complications that may significantly affect the quality of life in childhood and adulthood. Metabolomics together with epigenetics and proteomics is leading to the transformation of conventional medicine in personalized medicine, integrating multiple levels of information; they represent a challenge for promoting, maintaining and improving the health of populations through translational research.

## List of Non-Standard Abbreviations (Alphabetic Order)

AKI: Acute kidney injury
^1^H NMR: Proton nuclear magnetic resonance
AGA: Appropriate for gestational age
AKIN: Acute kidney injury network
BALF: Broncoalveolar lavage fluid
BUN: Blood urea nitrogen
CKD: Chronic kidney disease
CV: Coefficient of variation
DRF: Differential renal function
EGF: Epidermal growth factor
EMA: European medicines agency
ERK: Extracellular signal-regulated kinase
ESRD: End stage renal disease
ELBW: Extremely low birth weight
FDA: Food and drug administration
GC: Gas chromatography
GFR: Glomerular filtration rate
HAVCR-1: Hepatitis A virus cellular receptor-1
HMDB: Human metabolome data base
HN: Hydronephrosis
HNL: Human neutrophil lipocalin
IUGR: Intrauterine growth retardation
KIM-1: Kidney injury molecule-1
LBW: Low birth weight
LC: Liquid chromatography
Lcn-2: Lipocalin-2
LMW: Low molecular weight
MALDI-TOF: Matrix-assisted laser desorption and ionization time-of-flight
MAPK: Mitogen-activated protein kinase
MCP-1: Monocyte chemotactic peptide-1 ratio
MMP-9: Matrix metalloproteinase-9
MS: Mass spectrometry
NGAL: Neutrophil gelatinase-associated lipocalin
NICU: Neonatal intensive care unit
NIH: National Institute of Health
pRIFLE: Pediatric risk, injury, failure, end stage renal disease
PSTC: Predictive safety testing consortium
RDS: Respiratory distress syndrome
SCr: Serum creatinine
SD: Standard deviation
SELDI-TOF: Surface-enhanced laser desorption and ionization time-of-flight
TIM-1: T cell immunoglobulin mucin domains-1
uNGAL: Urine NGAL
UPJO: Ureteropelvic junction obstruction
VLBW: Very low birth weight.
